# Experiences of Dementia Diagnostic Disclosure Communication: A Qualitative Study of Patients and Caregivers

**DOI:** 10.1093/geroni/igaf122.2579

**Published:** 2025-12-31

**Authors:** Joanna Paladino, Heily Chavez Granados, Jade Connor Eruchalu, Ana-Maria Vranceanu, Deborah Blacker, Christine Ritchie

**Affiliations:** Massachusetts General Hospital, Boston, Massachusetts, United States; Massachusetts General Hospital, Boston, Massachusetts, United States; Massachusetts General Hospital, Boston, Massachusetts, United States; Massachusetts General Hospital, Boston, Massachusetts, United States; Massachusetts General Hospital, Boston, Massachusetts, United States; Massachusetts General Hospital, Boston, Massachusetts, United States

## Abstract

Clinician communication at the time of a dementia diagnosis inadequately addresses patient and caregiver needs. We aimed to characterize the disclosure experiences of patients and caregivers affected by dementia to identify communication domains that will be incorporated into the co-design of a diagnostic disclosure communication intervention. We conducted thematic analysis of individual interviews of patients and caregivers using a conceptual framework for person-centered communication. Participants included 6 patients with dementia and 15 caregivers of persons with dementia recruited from the community (n = 21; n = 17 female (81%); n = 13 Caucasian (61%); n = 4 Black or African American (19%); n = 4 Latino/a (19%); n = 2 Asian). We identified five themes. First, perceptions of respectful or disrespectful communication affects the relationship with clinicians and contributes to positive or negative experiences before, during, and after disclosure of a dementia diagnosis. Second, participants described the emotional impact of sudden or unsupported disclosures, in which they felt unprepared to receive the news or emotionally abandoned after diagnosis. Third, the absence of, or ambiguity around, a definitive dementia diagnosis contributes to distress and feeling dismissed by clinicians. Fourth, mixed responses to recommendations after disclosure reveals the need for a personalized care planning process. Fifth, considerations around the timing of prognostic communication and advance care planning are necessary to meet the needs of individuals with different emotional readiness, information preferences, and cultural beliefs. Dementia diagnostic disclosure would benefit from a tailored, structured, and incremental approach that prioritizes respectful communication, emotional support, and personalized care planning to meet the needs of patients and caregivers.

